# Spatial heterogeneity of heavy metals in contaminated soil using hyperspectral inversion models: a case study of Dongting lake region, south-central China

**DOI:** 10.1038/s41598-025-10313-6

**Published:** 2025-10-09

**Authors:** Gong Cheng, Xingwang Zhou, Meiqing Ding, Buqing Wang, Lingyi Liao

**Affiliations:** 1 Key Laboratory of Metallogenic Prediction of Nonferrous Metals and Geological Environment Monitoring, Ministry of Education, School of Geosciences and Info-Physics, Central South University, Changsha, 410083 China; 2 Hunan Key Laboratory of Remote Sensing Monitoring of Ecological Environment in Dongting Lake Area, Hunan Natural Resources Affairs Center, Changsha, China; 3 School of Traffic and Transportation Engineering, Changsha University of Science & Technology, Changsha, 410004 China; 4 Changsha Natural Resources Comprehensive Investigation Center, China Geological Survey, Changsha, 410625 China

**Keywords:** OHS, Soil heavy metals, Remote sensing, Inversion, Deep learning, Environmental sciences, Solid Earth sciences

## Abstract

Heavy metal pollution in soil seriously threatens ecosystem and human health. However, traditional monitoring methods usually rely on intensive sampling, which is costly and difficult to be extended to large regional scales. Based on Orbita Hyperspectral Satellites (OHS) imagery and 175 sample sets out of 1589 samples, Multiple Linear Regression (MLR), Partial Least Squares Regression (PLSR), Support Vector Machine Regression (SVM), Back Propagation Neural Network (BPNN), and Convolutional Neural Network (CNN) models were constructed to predict eight elements (As, Cd, Cr, Cu, Hg, Ni, Pb, Zn). To explore the feasibility of using a small number of samples to invert the distribution trend of heavy metals in a large area. The results show among the above eight elements, the retrieval of Pb is the best, with the R^2^ of BPNN and CNN reaches 0.80. BPNN and CNN achieves the optimal inversion of As, Cd and Pb. MLR and PLSR has the best accuracy in Cr and Cu, Hg, Ni and Zn. In addition, the distribution trends of 8 heavy metals retrieved from a small number of samples were basically consistent with the interpolation maps of 1589 samples, indicating that it is completely feasible to use a small number of samples to retrieve the distribution trends of heavy metals in large areas. This study provides important technical support for regional soil pollution prevention and control, and has significant application value and promotion potential.

## Introduction

In recent years, soil heavy metal pollution has become one of the important ecological problems globally^1–4^. The Dongting Lake region, which is identified as the youngest, most distinctive, and fastest soil-forming estuarine delta worldwide, is the only most intact, typical, and young wetland ecosystem in the warm temperate zone, with abundant biological resources^5–7^. High-intensity human activities such as mineral development, fossil fuel combustion, land use practices, petroleum exploitation, and agricultural cultivation have caused serious soil heavy metal pollution as a result of the Dongting Lake region’s economic growth^8–11^. Therefore, how to quickly and accurately detect soil heavy metal pollution areas and assess the degree of pollution is the key to carrying out soil pollution monitoring and environmental management.

The traditional method of obtaining soil heavy metal content requires researchers to collect soil samples from the field and send them to the laboratory for chemical composition analysis^12–15^. This method has the problems of a small amount of data, high cost, long cycle, and limited research scope, which makes it difficult to meet the rapid and large area determination of soil heavy metal content. With the continuous development of remote sensing technology and the increase of remote sensing data, it is possible to monitor the content of heavy metals in soil in a large range. Remote sensing technology, with the advantages of efficiency, large coverage, environmental friendliness, and non-destructive to soil, has attracted wide attention in the research of soil heavy metal content prediction. Currently, most soil heavy metal retrieval based on hyperspectral data use the measured hyperspectral data^16–20^. The measured hyperspectral data has the characteristics of high spectral resolution and continuity, and can usually achieve high inversion accuracy. However, the process of acquiring and analyzing hyperspectral data is complex and costly, which is difficult to adapt to the inversion of heavy metal content in large areas of soil. Therefore, hyperspectral images with wide coverage, low cost, and fast acquisition speed are more suitable for the monitoring of soil heavy metal content in a wide range, and many studies have achieved remarkable results in this field^21–24^. Currently, linear regression^21,22,25,26^ and traditional machine learning^23,27–29^ methods are mainly used to retrieve soil heavy metal content from hyperspectral images, while deep learning^24,30^ especially convolutional neural networks, have relatively few applications in this field, and there is a lack of comparative studies of multiple heavy metal elements and multiple models.

At present, in the authenticity test of quantitative inversion, the mainstream evaluation indicators include RMSE, R², MRE, ME, and RPD based on mathematical statistical analysis^22–24^ and comparing the model inversion results with the interpolated plot of the sample set^31^. It mainly evaluates the accuracy of the model through statistical analysis of a small number of samples selected from the sample set. However, Quantitative inversion of soil element content aims to infer the distribution trend of soil element content in a large range through a small range and a small number of samples. These evaluation methods are difficult to judge the applicability of the model in unknown large-scale regions.Studies that focus on predicting broader spatial trends of soil heavy metal distribution using only sparse samples from localized areas remain limited, and further research in this area is urgently needed.

Based on the research background, this study used Orbita Hyperspectral Satellites (OHS) imagery and 175 samples from five sub-regions extracted from 1589 sample sets to establish Multiple Linear Regression (MLR), Partial Least Squares Regression (PLSR), Support Vector Machine Regression (SVM), Back Propagation Neural Network (BPNN), and Convolutional Neural Network (CNN) models. The contents of eight heavy metal elements (As, Cd, Cr, Cu, Hg, Ni, Pb, Zn) in the soil of the southeastern edge of Dongting Lake were inverted, and the inversion effect of the optimal model for each element was compared with the interpolation effect of 1589 samples. This study aims to explore the feasibility of retrieving a large range of soil heavy metal distribution trends from a small number of samples in a small area.

## Overview of the study area

**Fig. 1:**
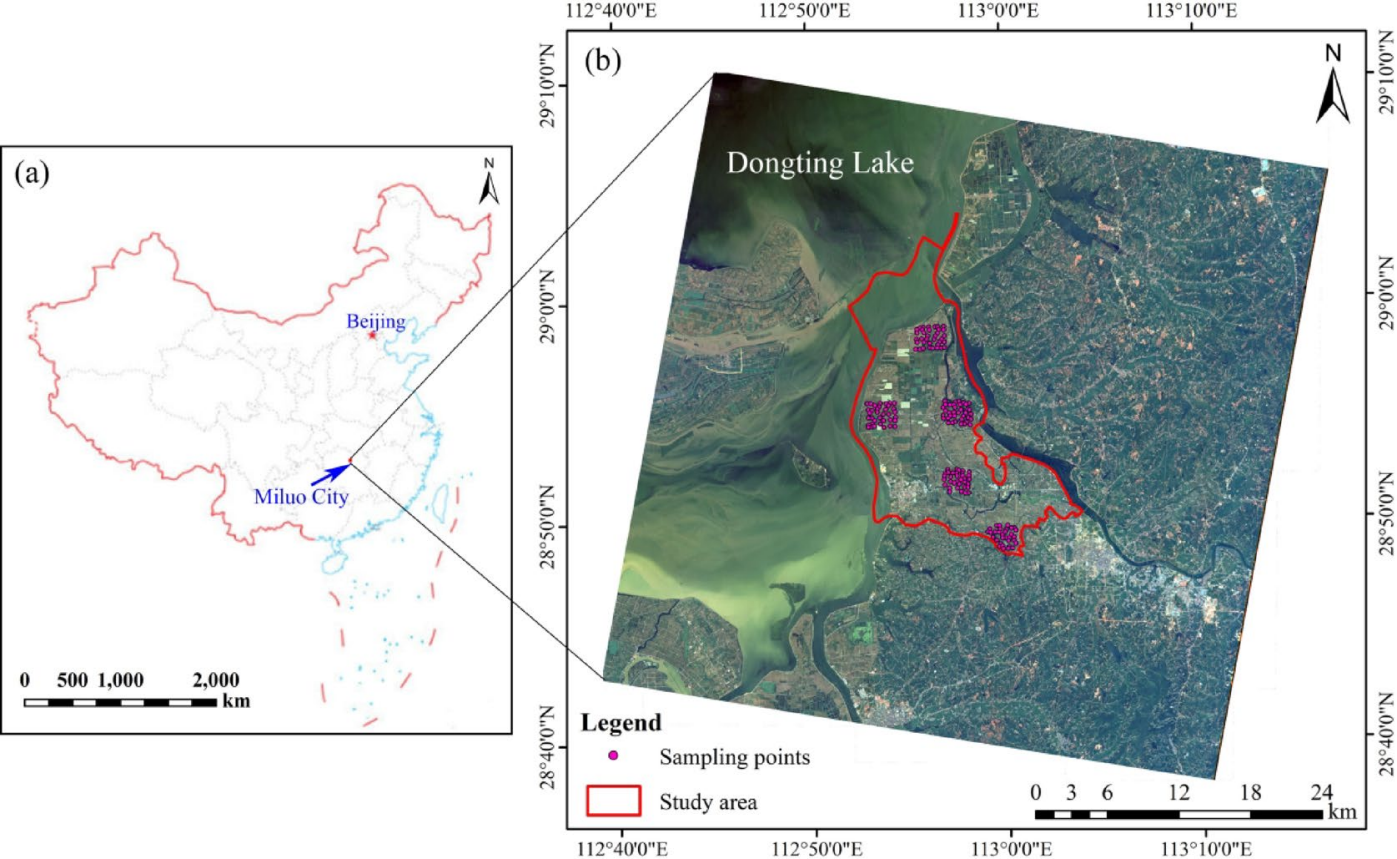
(**a**) Simplified map of China; (**b**) Location of study area and distribution map of Modeling sample points (The satellite images are the 13, 6, and 2 bands of the OHS).

The study area is located on the southeast edge of Dongting Lake and the northwest of Miluo City, China (28°48 ‘23 “−29°03’ 56” N, 112°52 ‘03 “−113°03’ 55” E), including Yingtian Town, Fenghuang Township, the northwest of River Town and the west of Quzici Town, with a total area of about 186 km^2^ (Fig. 1). It is situated in a subtropical humid climate, with well-defined four seasons and concentrated rainfall. The study area is flat and mainly cultivated, and contains a small amount of building land and forest land. The soil is mainly formed by river and lake sediments, mostly paddy soil, with a strong viscous texture, mostly clay and loam. The water system in this area is developed, including Xiangjiang River in the west, Miluo River in the east, and East Dongting Lake in the north^32^.

## Materials and methods

### Soil sample data collection and processing

**Fig. 2:**
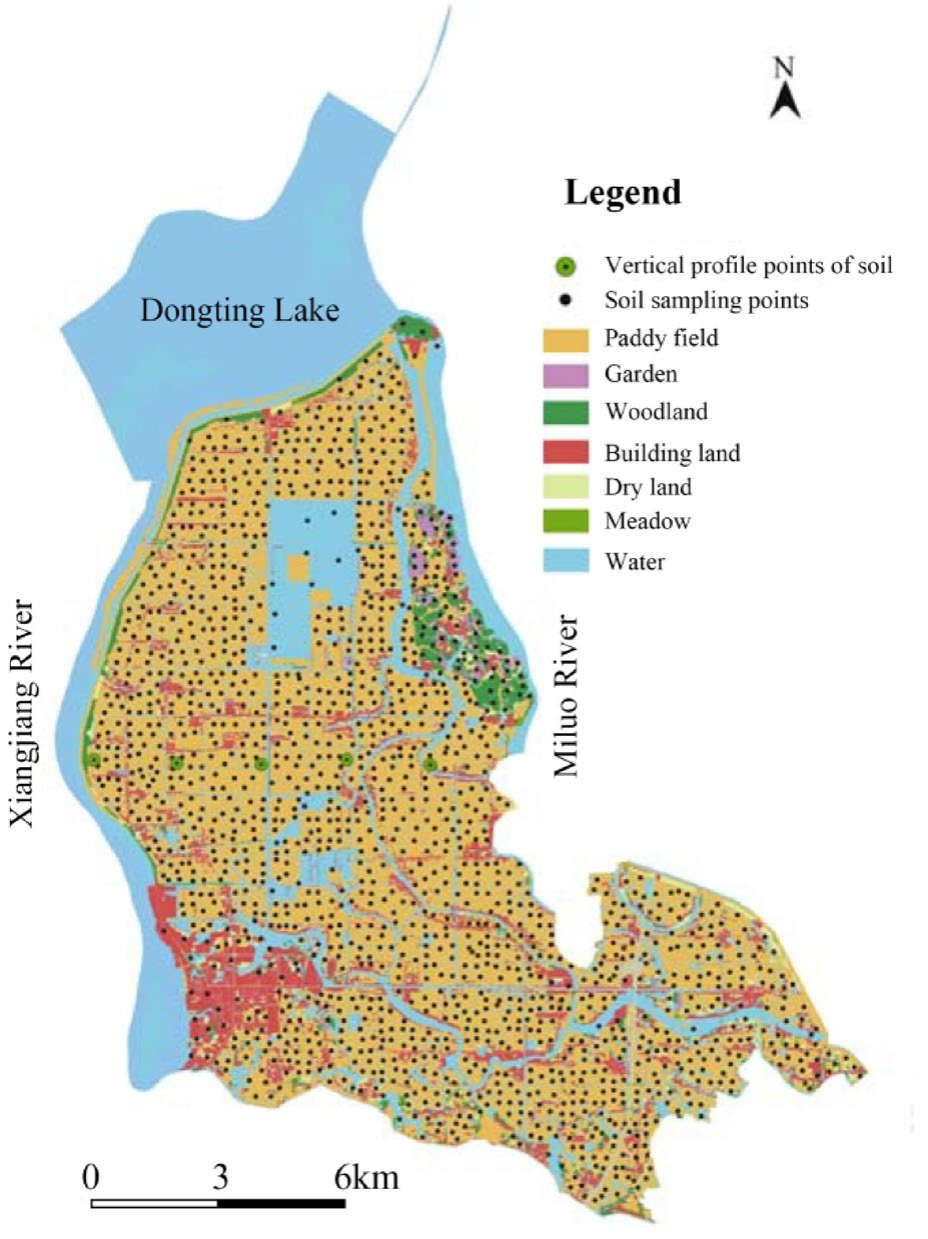
Distribution map of geochemical sampling points in the study area^32^.

**Fig. 3:**
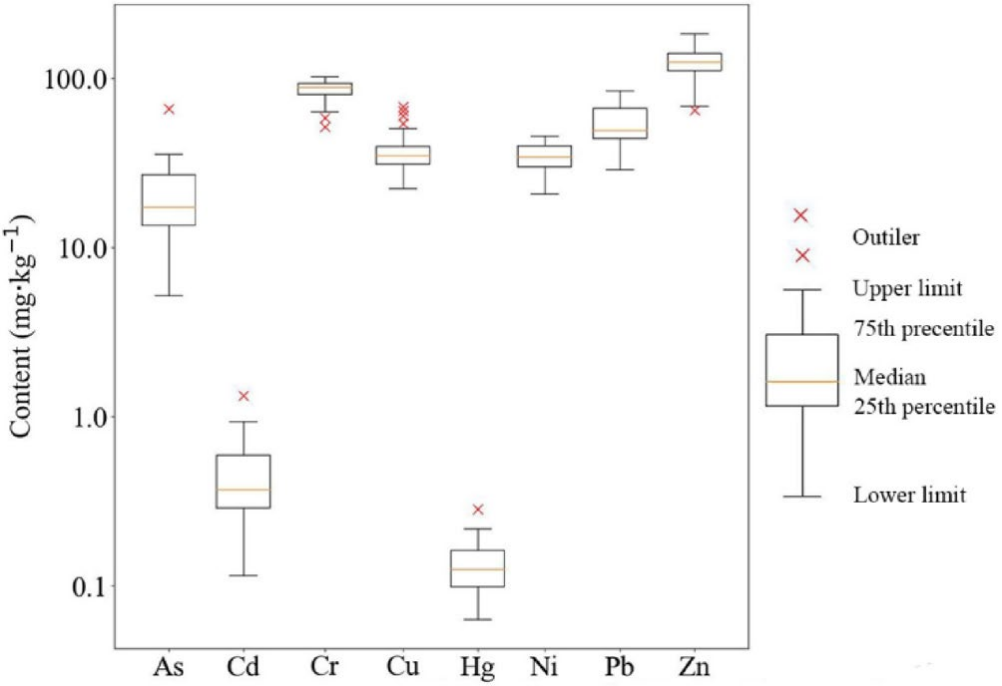
Box plot of soil heavy metal conten.

In this research work, soil data were collected from April to August 2020. The “×” or “S” sampling method was used for sampling with a sampling depth of 0–20 cm. Each sample was about 2 kg and consisted of 4 to 6 sub-samples mixed in equal proportions. A total of 1589 soil samples were collected and mainly distributed in paddy fields, followed by forests, gardens, grasslands, construction land, and water bodies (Fig. 2). After sample collection, the dead branches and leaves in the samples were removed and processed by sun drying, crushing, grinding, screening, sealing and chemical testing^32^. A total of 175 samples from 5 sub-regions were randomly selected from 1589 soil samples. In this experiment, the heavy metal geochemical data of these samples were used for quantitative remote sensing modeling. Because regression models are sensitive to extreme values (outliers), these extreme data points can have a large impact on the model. Therefore, the extreme values are clearly divided by the box plot, thus enabling the model to fit the main trends of the data more accurately. Except for multiple outliers of Cu and Cr elements, the anomalies of other elements are not significant (Fig. 3). And the content of 8 elements after removing outliers was statistically analyzed (Table 1).

**Table 1:** Table of statistical analysis of geochemical data of heavy metals in soil samples.

Element	Sample size (number)	Maximum/(10^−6^)	Minimum/(10^−6^)	Mean/(10^−6^)	Standard deviation	Variable coefficient/%
As	174	35.453	5.172	19.836	7.239	36.496
Cd	174	0.941	0.115	0.446	0.201	45.066
Cr	173	102.000	63.600	87.022	8.497	9.764
Cu	171	50.600	22.300	35.575	5.788	16.269
Hg	174	0.217	0.063	0.129	0.035	27.359
Ni	175	45.600	20.700	34.709	5.576	16.585
Pb	175	84.700	29.000	54.538	14.259	26.145
Zn	174	184.000	68.600	124.009	23.230	18.733

### Hyperspectral data acquisition and preprocessing

Remote sensing image data are collected by the Orbita HyperSpectral satellite by Zhuhai Orbital Aerospace Science & technology Co., Ltd. The OHS image has 32 bands with a spatial resolution of 10 m, a spectral range of 400–1000 nm, and a spectral resolution of 2.5 nm. The width is greater than 150 km^33^. Currently, there are eight hyperspectral satellites in orbit, with a revisit period of six days for each satellite. The eight hyperspectral satellites can cover the globe for 2.5 days and revisit a specific area for one day^34^. The scene number of the OHS remote sensing data obtained in this study is HCM2_20200803200423_0008_L1B_CMOS2, which was acquired by the OHS-2 C satellite on August 3, 2020 and close to the time of ground sample acquisition, ensuring the synchronization of sampling and remote sensing data, thereby reducing the error sources in quantitative inversion. The selected images are clear and cloud-free, which can meet the requirements of quantitative retrieval of soil heavy metals by remote sensing (Table 2).

**Table 2:** OHS image parameters.

Parameter	Parameter values	Parameter	Parameter values
Imaging time	2020.08.03	Spectral resolution	2.5 nm
Number of bands	32	Spectral range	400–1000 nm
Spatial resolution	10 m	Cloud cover	0%

ENVI 5.3 software is used to preprocess the OHS data. Firstly, the surface reflectance of the study area is obtained by radiometric calibration and FLAASH atmospheric correction of the OHS images. Then, the RPC file and ASTER GDEM data built into the OHS image are used to correct the image data. Finally, the control points are used to geo-register them^35^.

### Correlation analysis

When using machine learning methods for regression fitting, inputting too much data will lead to slow model convergence and affect model performance. Therefore, this paper chose to use the Spearman correlation coefficient method to analyze the correlation between each spectral feature and the measured soil heavy metal content, and selected the features with strong correlation to establish the regression model. Spearman correlation analysis is a nonparametric statistical method for assessing the rank association between two variables, especially when the data does not meet the normal distribution. It can well measure the linear and nonlinear correlation between two variables X and Y, and its value is between − 1 and 1. The stronger the correlation of two variables is, the closer its absolute value is to 1. Its definition is as follows:

1 $$\:{d}_{i}=R\left(x\right)-R\left(y\right)$$

2 $$\:{r}_{s}=1-\frac{6\sum\:{d}_{i}^{2}}{n({n}^{2}-1)}$$

where: $\:R\left(x\right)$and $\:R\left(y\right)$ are the rank of $\:{x}_{i}$ and $\:{y}_{i}$, respectively; $\:{d}_{i}$ is the rank difference between the data points. *n* is the number of observations; $\:{r}_{s}$ represents the Spearman correlation value.

### Model establishment

In this study, the Spearman correlation analysis method was first used to analyze the correlation of 32 bands and 8 heavy metal elements of OHS remote sensing data to screen the characteristic bands. First, we use the selected feature bands as input variables, then we select five models including linear models like MLR and PLSR, traditional machine learning models such as SVM, and the deep learning model involving BPNN and CNN to retrieve soil heavy metal content by model inversion. The influence of different inversion algorithms on soil heavy metal content retrieval and the potential of deep learning in soil heavy metal inversion are discussed.

By selecting the above five types of models we considered and covered different modeling strategies from traditional statistical methods to modern machine learning algorithms. By comparing the performance differences between linear and nonlinear, shallow and deep models, the adaptability of different algorithms to data characteristics is systematically evaluated.

MLR is a multivariate statistical regression method that tests the relationship between a single dependent variable and a set of independent variables^36,37^. PLSR is a multivariate regression analysis technique, that introduces principal component analysis on the basis of traditional least squares regression analysis. It uses principal component analysis to extract the principal components of the independent variables and builds the model through stepwise regression^38,39^. Based on kernel statistics theory, SVMR improves the generalization ability of the model by finding the minimum structural risk. SVMR can balance the complexity and learning ability of the model with limited sample information and has advantages in establishing small sample, nonlinear, and high-dimensional models^40,41^. The relevant hyperparameters of PLSR and SVM are selected based on the training set using cross-validation and grid search algorithms, the hyperparameter search range is shown in Table 3.

**Table 3:** SVM and RF hyperparameter grid search range.

Models	Hyperparameter	Search range
PLSR	n_components	1–32
SVM	kernel	RBF
	C	0.001, 0.01, 0.1, 1–200
	gamma	0.001, 0.01, 0.1, 1–200

**Fig. 4:**
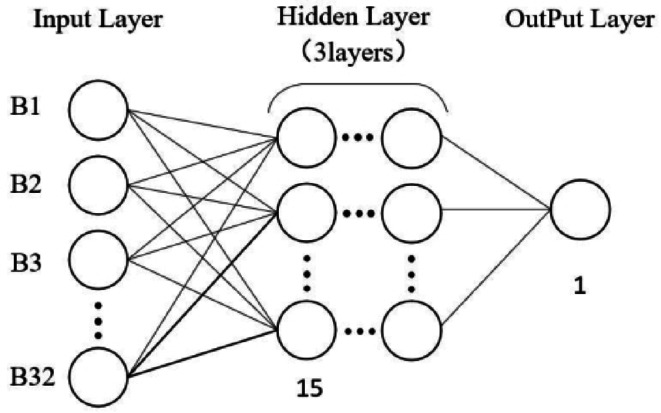
Diagram of the BPNN structure.

BPNN was introduced by Rumelhart and others in 1986 and is currently one of the most widely used neural networks^42^. The BPNN consists of an input layer, a hidden layer, and an output layer, each of which consists of multiple neurons. The training process of BP neural network is mainly divided into two stages: forward propagation and back propagation. The back-propagation algorithm uses the idea of gradient descent to update the weights by calculating the partial derivative of the loss function with respect to the connection weights. The goal of BPNN is to minimize the error between the actual output value and the desired output value through gradient descent, which has a strong nonlinear prediction ability^29,43^. In this study, the number of input and output nodes is set to 32 and 1, respectively, the number of hidden layers is 3, and the number of nodes is set to 15. The activation function of hidden layer is the tanh function, and the activation function of output layer is linear (Fig. 4). The Adaptive Time Estimation algorithm (Adam) was used for optimization.

**Fig. 5:**
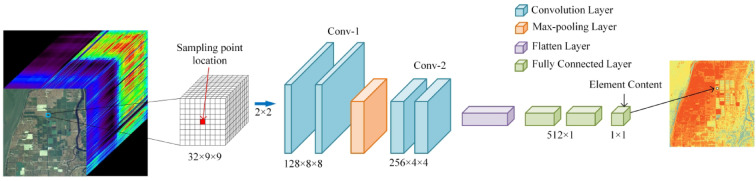
Diagram of the CNN structure (The satellite images show the 32 bands of the OHS).

CNN is a kind of feedforward neural network, that uses forward propagation and back propagation algorithms for training and network optimization, and finally obtains the output value^44^. CNN usually consists of multiple convolutional layers, pooling layers, and fully connected layers^45^. A convolutional layer is the core part of CNN, which performs convolution operations on input data to extract features through the convolution kernel. Compared with the fully connected network method, the convolutional layer can efficiently extract features and reduce the number of model parameters. Each convolutional layer performs a convolution operation on the features of the previous layer, and then feeds the result into the activation function to extract high-dimensional features^46,47^. The input of CNN is to take the raster image corresponding to the sample point as the pixel center, and extract the surrounding 9 × 9 raster matrix. The convolutional layer consists of four layers, and every two convolutional layers are followed by a Max pooling layer. The first convolutional layer has a kernel size of 2 × 2 to make the matrix size even in order to connect the pooling layers, and the other convolutional layers have a kernel size of 3 × 3. To avoid overfitting and enhance the generalization ability of the model, a dropout layer is connected to the flattening layer. The convolutional layer is followed by two fully connected layers with 512 nodes and one node in the output layer. The activation function of the output layer is a linear function, and Relu is selected as the activation function of the other layers, which is also optimized by the Adaptive Time Estimation algorithm (Adam) (Fig. 5).

### Precision evaluation

In this study, 70% of the samples were randomly selected as the training set, the remaining 30% of the samples were selected as the validation set, and the coefficient of determination (R^2^), Ratio of standard deviation of the validation set to standard error of prediction (RPD), Root Mean Square Error (RMSE), and Mean Absolute Error (MAE) index to evaluate the inversion accuracy of the established model^48,49^.

1 $$\:{R}^{2}=1-\frac{{\sum\:}_{i=1}^{N}{({y}_{m}-{y}_{p})}^{2}}{{\sum\:}_{i=1}^{N}{({y}_{m}-{\overline{y}}_{m})}^{2}}$$

2 $$\:RPD=\frac{SD}{RMSE}=\sqrt{\frac{N{\sum\:}_{i=1}^{N}{({y}_{m}-{\overline{y}}_{m})}^{2}}{(N-1){\sum\:}_{i=1}^{N}{({y}_{m}-{y}_{p})}^{2}}}$$

3 $$\:RMSE=\sqrt{\frac{{\sum\:}_{i=1}^{N}{({y}_{m}-{y}_{p})}^{2}}{N}}$$

4 $$\:MAE=\frac{1}{N}{\sum\limits_{i=1}^{N}}\left|{y}_{m}-{y}_{p}\right|$$

where, *y*_*m*_ is the measured value of element content; *y*_*p*_ is the predicted value of the element content; $\:{\overline{y}}_{m}$ is the average value of the measured element content. *N* is the number of samples. *SD* is the standard deviation.

## Results

### Correlation analysis results

According to the Spearman correlation analysis results in Table 4, the 32 bands (B1-B32) of OHS remote sensing image showed significant negative correlation with 8 soil heavy metal elements (As, Cd, Cr, Cu, Hg, Ni, Pb, Zn), and the correlation strength showed obvious rules with the change of band.

**Table 4:** Spearman correlation was used to analyze the results.

	As	Cd	Cr	Cu	Hg	Ni	Pb	Zn
B1 (443 nm)	–0.355**	–0.394**	–0.299**	–0.370**	–0.338**	–0.368**	–0.478**	–0.419**
B2 (466 nm)	–0.277**	–0.323**	–0.233**	–0.299**	–0.259**	–0.288**	–0.393**	–0.342**
B3 (490 nm)	–0.166*	–0.215**	–0.146	–0.211**	–0.156*	–0.182*	–0.271**	–0.231**
B4 (500 nm)	–0.105	–0.159*	–0.108	–0.146	–0.108	–0.127	–0.213**	–0.172*
B5 (510 nm)	–0.099	–0.156*	–0.105	–0.138	–0.100	–0.121	–0.205**	–0.164*
B6 (531 nm)	–0.106	–0.165*	–0.094	–0.138	–0.098	–0.112	–0.204**	–0.168*
B7 (550 nm)	–0.094	–0.165*	–0.089	–0.133	–0.091	–0.101	–0.184*	–0.164*
B8 (560 nm)	–0.046	–0.131	–0.054	–0.091	–0.059	–0.056	–0.141	–0.125
B9 (580 nm)	–0.015	–0.103	–0.037	–0.054	–0.031	–0.029	–0.104	–0.088
B10 (596 nm)	0.011	–0.080	–0.026	–0.032	–0.009	–0.009	–0.078	–0.064
B11 (620 nm)	0.020	–0.066	–0.023	–0.023	–0.004	–0.003	–0.066	–0.055
B12 (640 nm)	0.019	–0.068	–0.030	–0.025	–0.008	–0.006	–0.068	–0.057
B13 (665 nm)	0.030	–0.064	–0.017	–0.019	–0.007	0.000	–0.065	–0.053
B14 (670 nm)	0.027	–0.069	–0.024	–0.023	–0.010	–0.007	–0.070	–0.057
B15 (686 nm)	–0.019	–0.113	–0.063	–0.069	–0.047	–0.057	–0.124	–0.112
B16 (700 nm)	–0.230**	–0.294**	–0.199**	–0.262**	–0.233**	–0.267**	–0.354**	–0.327**
B17 (709 nm)	–0.431**	–0.413**	–0.318**	–0.439**	–0.390**	–0.443**	–0.538**	–0.493**
B18 (730 nm)	–0.461**	–0.401**	–0.315**	–0.443**	–0.421**	–0.460**	–0.547**	–0.491**
B19 (746 nm)	–0.445**	–0.374**	–0.293**	–0.428**	–0.397**	–0.440**	–0.519**	–0.470**
B20 (760 nm)	–0.460**	–0.382**	–0.305**	–0.440**	–0.410**	–0.455**	–0.529**	–0.485**
B21 (776 nm)	–0.468**	–0.389**	–0.312**	–0.451**	–0.418**	–0.464**	–0.536**	–0.496**
B22 (780 nm)	–0.472**	–0.395**	–0.313**	–0.455**	–0.424**	–0.467**	–0.540**	–0.502**
B23 (806 nm)	–0.477**	–0.395**	–0.317**	–0.458**	–0.430**	–0.473**	–0.545**	–0.507**
B24 (820 nm)	–0.467**	–0.383**	–0.313**	–0.452**	–0.426**	–0.466**	–0.538**	–0.500**
B25 (833 nm)	–0.462**	–0.377**	–0.316**	–0.451**	–0.422**	–0.465**	–0.533**	–0.496**
B26 (850 nm)	–0.455**	–0.376**	–0.312**	–0.449**	–0.419**	–0.460**	–0.529**	–0.492**
B27 (865 nm)	–0.453**	–0.378**	–0.307**	–0.451**	–0.418**	–0.458**	–0.527**	–0.489**
B28 (880 nm)	–0.451**	–0.369**	–0.305**	–0.447**	–0.413**	–0.454**	–0.519**	–0.484**
B29 (896 nm)	–0.438**	–0.358**	–0.299**	–0.444**	–0.408**	–0.443**	–0.507**	–0.474**
B30 (910 nm)	–0.449**	–0.369**	–0.307**	–0.454**	–0.419**	–0.452**	–0.517**	–0.486**
B31 (926 nm)	–0.486**	–0.412**	–0.340**	–0.478**	–0.465**	–0.493**	–0.554**	–0.519**
B32 (940 nm)	–0.534**	–0.456**	–0.375**	–0.507**	–0.514**	–0.536**	–0.592**	–0.550**

The symbols * and ** indicate significant correlations at the 0.05 and 0.01 levels, respectively.

On the whole, B16-B32 (700–940 nm) showed the strongest negative correlation, and the band B32 (940 nm) had the highest correlation coefficient (–0.534 to –0.592) among all heavy metals, especially for Pb, which was − 0.592, indicating that this band had the best indication for heavy metal pollution. B1-B3 (443–490 nm) also showed moderate intensity correlation (− 0.166 to − 0.478), and B4-B15 (500–686 nm) correlation was significantly reduced, and some bands were even not statistically significant. Based on the results of Spearman correlation analysis, for eight heavy metal elements such as As, Cd, Cr, Cu, Hg, Ni, Pb and Zn, the characteristic bands with significant (*) or extremely significant (**) correlation coefficients were selected as modeling variables, and the inversion models of eight heavy metal contents were constructed.

### Analysis of soil heavy metal content estimated by different models

MLR, PLSR, SVMR, BPNN, and CNN models were used to retrieve the content of eight heavy metal elements in the soil of the study area, including As, Cd, Cr, Cu, Hg, Ni, Pb, and Zn. R^2^ and RMSE were used to evaluate the inversion accuracy of the above different heavy metal elements and different models (Table 5).

**Table 5:** Comparison of retrieval accuracy for soil heavy metal train and test sets.

Element	*R* ^2^					RPD					RMSE					MAE				
	MLR	PLSR	SVM	BP	CNN	MLR	PLSR	SVM	BP	CNN	MLR	PLSR	SVM	BP	CNN	MLR	PLSR	SVM	BP	CNN
As	0.687	0.686	0.708	0.720	0.740	1.804	1.801	1.868	1.908	1.980	4.123	4.129	3.981	3.899	3.756	3.459	3.466	3.191	3.134	3.001
Cd	0.566	0.544	0.596	0.632	0.651	1.533	1.497	1.588	1.663	1.709	0.148	0.151	0.143	0.136	0.133	0.119	0.126	0.111	0.105	0.102
Cr	0.371	0.400	0.310	0.307	0.300	1.273	1.304	1.216	1.213	1.207	6.936	6.774	7.266	7.282	7.319	5.526	5.449	5.689	5.897	5.880
Cu	0.381	0.284	0.322	0.300	0.212	1.283	1.193	1.226	1.206	1.137	4.617	4.965	4.593	4.911	5.210	3.312	3.582	3.552	3.609	3.869
Hg	0.528	0.511	0.473	0.509	0.463	1.470	1.444	1.391	1.441	1.378	0.026	0.026	0.027	0.026	0.027	0.021	0.022	0.021	0.021	0.022
Ni	0.649	0.660	0.640	0.526	0.639	1.703	1.732	1.683	1.466	1.679	3.571	3.511	3.256	4.147	3.622	2.983	2.903	2.555	3.316	3.006
Pb	0.755	0.764	0.680	0.801	0.791	2.042	2.078	1.785	2.263	2.204	7.521	7.390	8.605	6.785	6.968	5.741	5.741	6.728	5.474	5.423
Zn	0.639	0.642	0.511	0.609	0.564	1.681	1.687	1.443	1.615	1.529	14.61	14.56	17.02	15.21	16.07	11.36	11.31	12.80	11.62	12.57

In general, the retrieval accuracy of soil Pb content was the best, and the R^2^ of the five models in the test set was above 0.68, and the R^2^ of BPNN and CNN was about 0.8, and the RPD of BPNN and CNN was about 2.2. However, Cr and Cu perform poorly in inversion, with both test set R^2^ below 0.4. BPNN has the best performance in Pb inversion. However, in Cr, Ni, Hg, and Cu retrieval, the test set accuracy of BPNN is not significantly improved compared with the linear regression model, and even in Cu inversion, its performance is worse than MLR. CNN achieves the optimal inversion of As and Cd, but the inversion accuracy of the test set is close to that of BPNN, and the inversion accuracy of Cr, Cu, Hg, and Zn is poor (Table 5). Although the inversion accuracy of CNN is better than that of BPNN in some elements, the inversion effect of BPNN is more stable. In addition, the inversion accuracy of MLR and PLSR is close, and the optimal retrieval of Cr, Cu, Hg and Ni is achieved. In general, the inversion accuracy of SVM is the worst, except that it is slightly better than MLR and PLSR in the retrieval of As and Cd, and is lower than that of the linear regression model in the other 6 soil heavy metal retrievals (Table 5).

### Analysis of soil heavy metal content estimated by different models

**Fig. 6:**
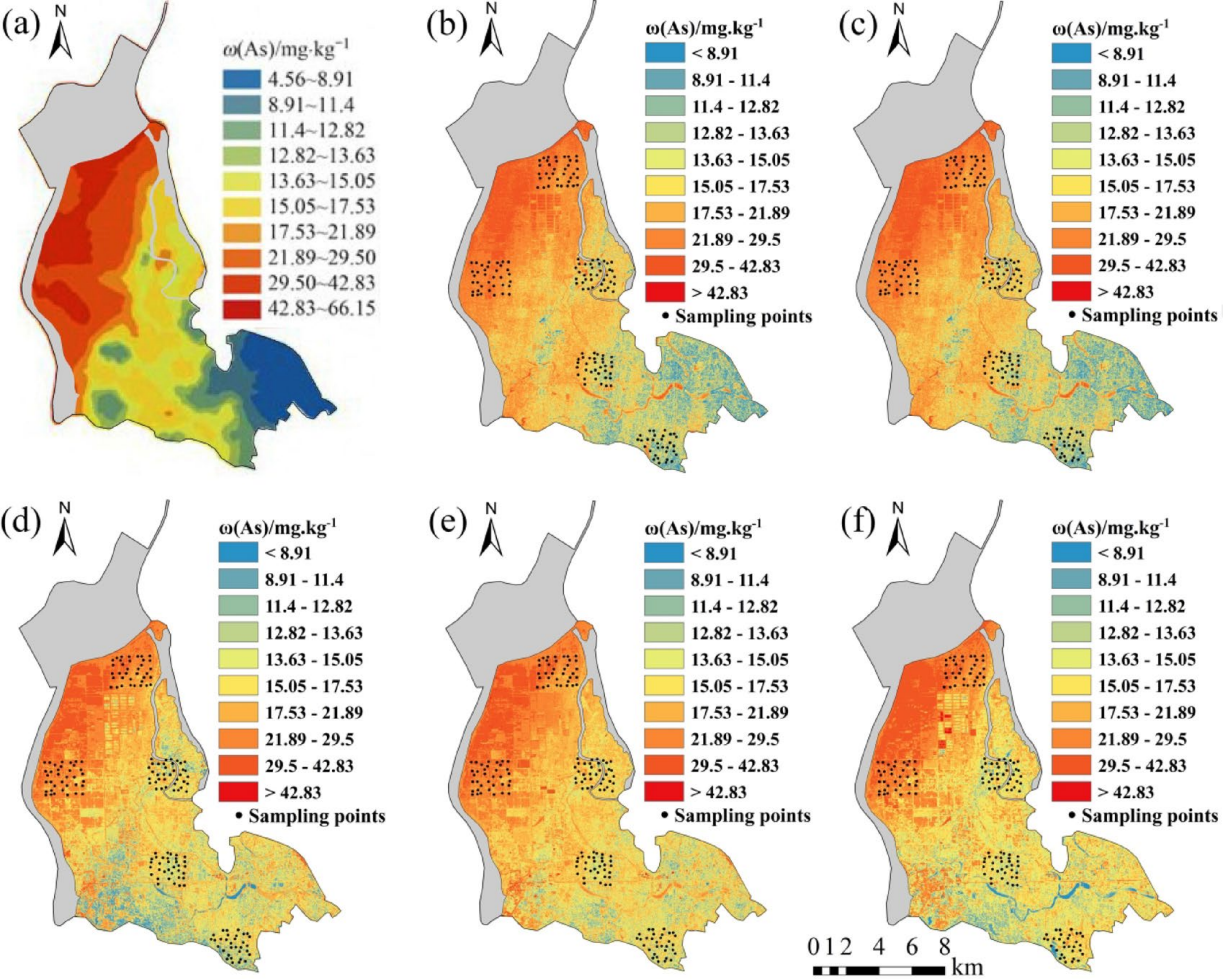
Soil As content distribution map: (**a**) Interpolation; (**b**) MLR; (**c**) PLSR; (**d**) SVM; (**e**) BP; (**f**)CNN.

**Fig. 7:**
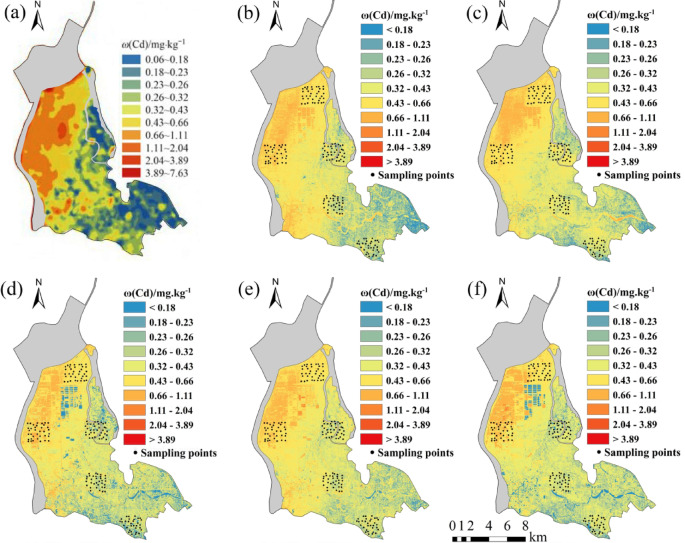
Soil Cd content distribution map: (**a**) Interpolation; (**b**) MLR; (**c**) PLSR; (**d**) SVM; (**e**) BP; (**f**)CNN.

**Fig. 8:**
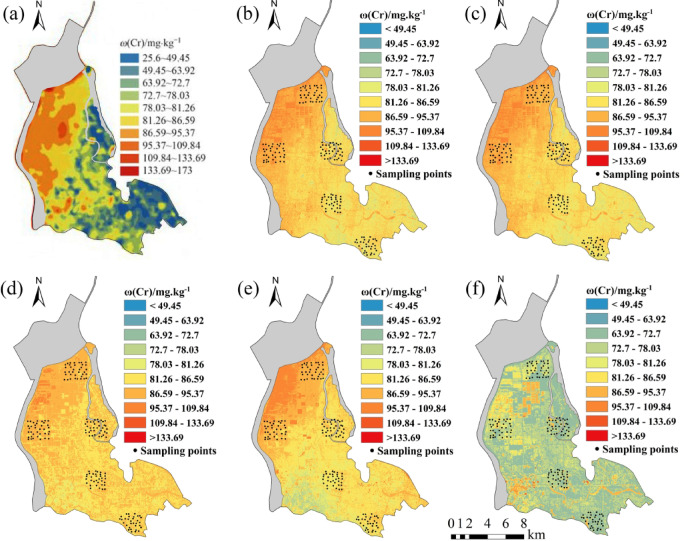
Soil Cr content distribution map: (**a**) Interpolation; (**b**) MLR; (**c**) PLSR; (**d**) SVM; (**e**) BP; (**f**) CNN.

**Fig. 9:**
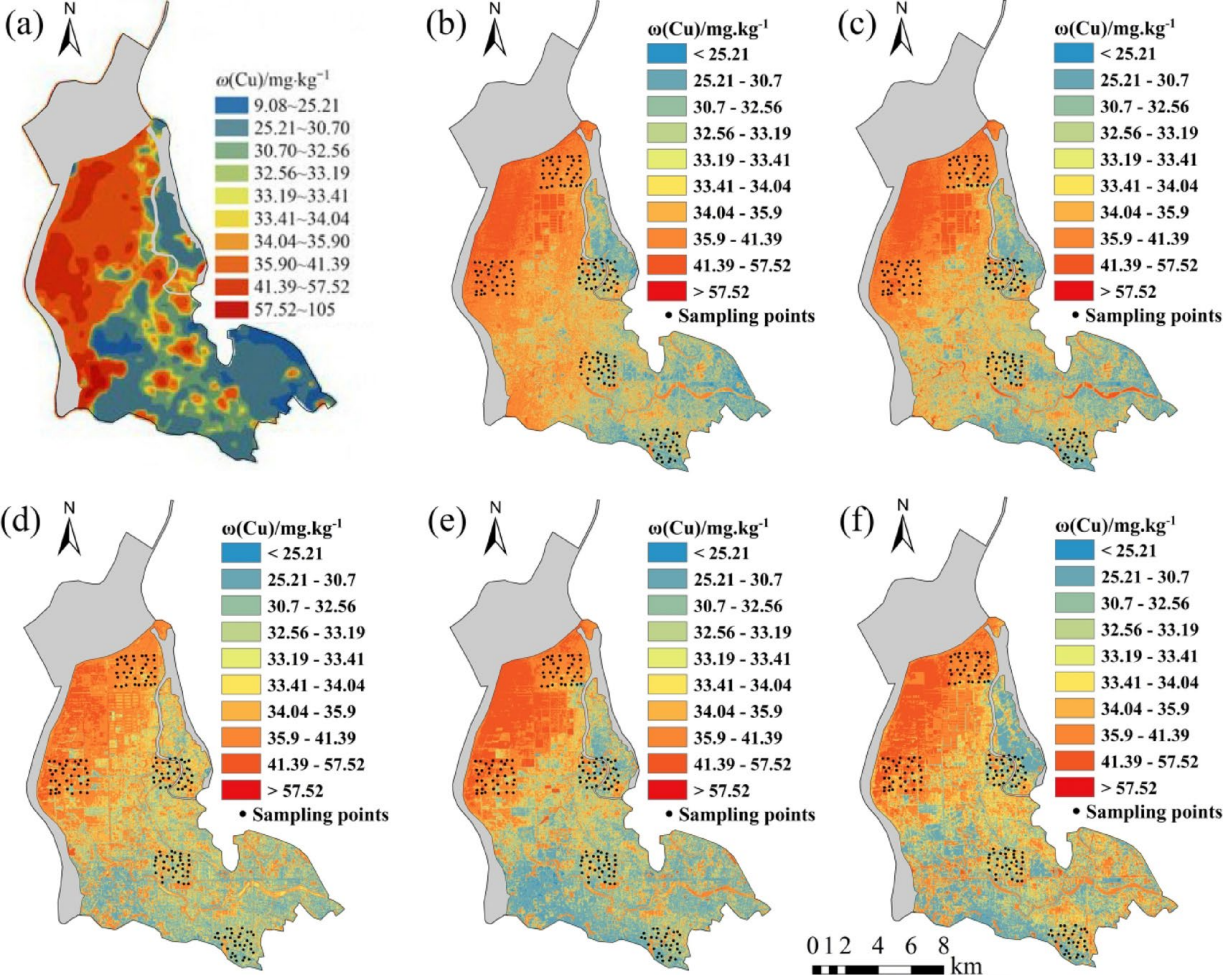
Soil Cu content distribution map: (**a**) Interpolation; (**b**) MLR; (**c**) PLSR; (**d**) SVM; (**e**) BP; (**f**)CNN.

**Fig. 10:**
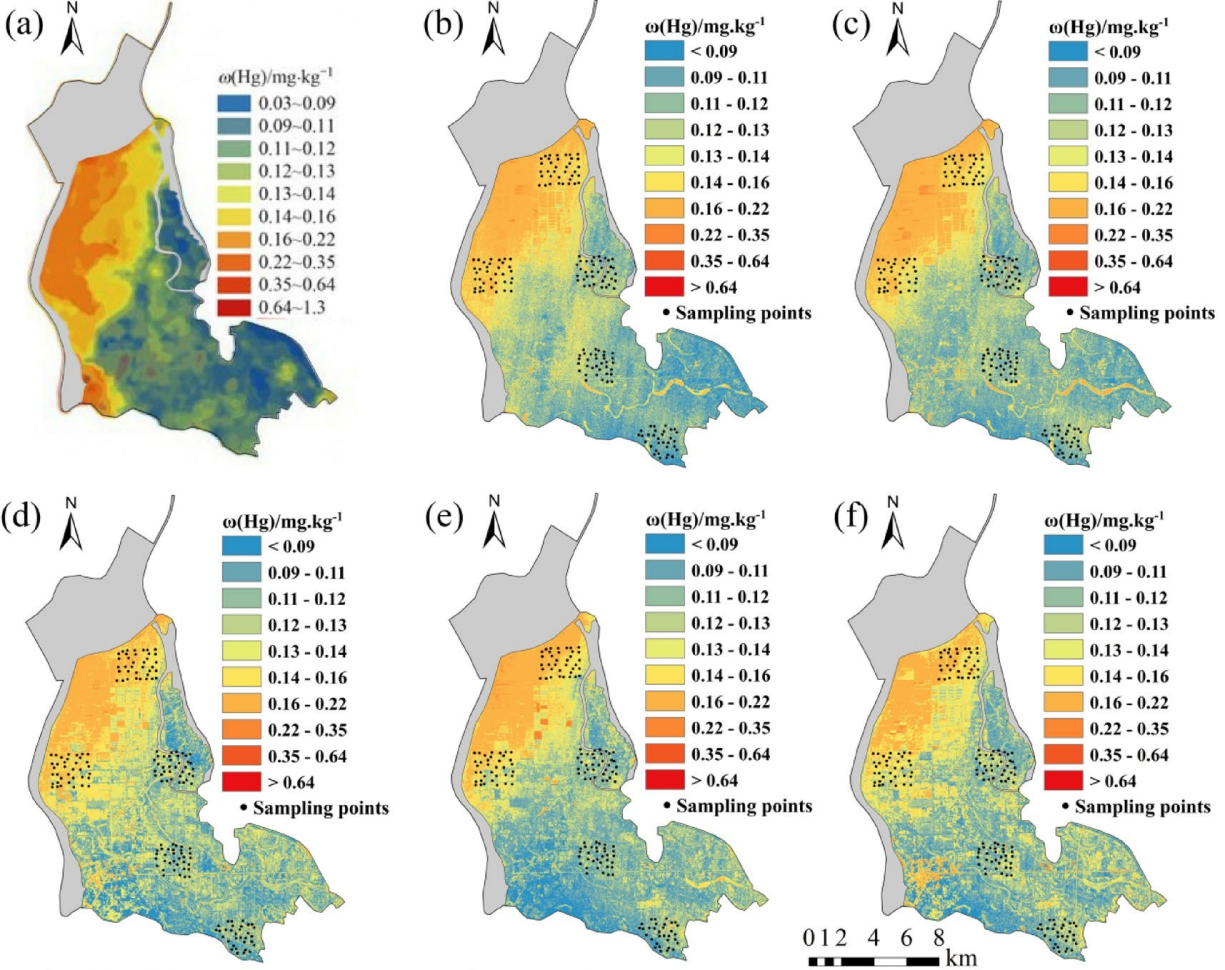
Soil Hg content distribution map: (a) Interpolation; (b) MLR; (c) PLSR; (d) SVM; (e) BP; (f)CNN.

**Fig. 11:**
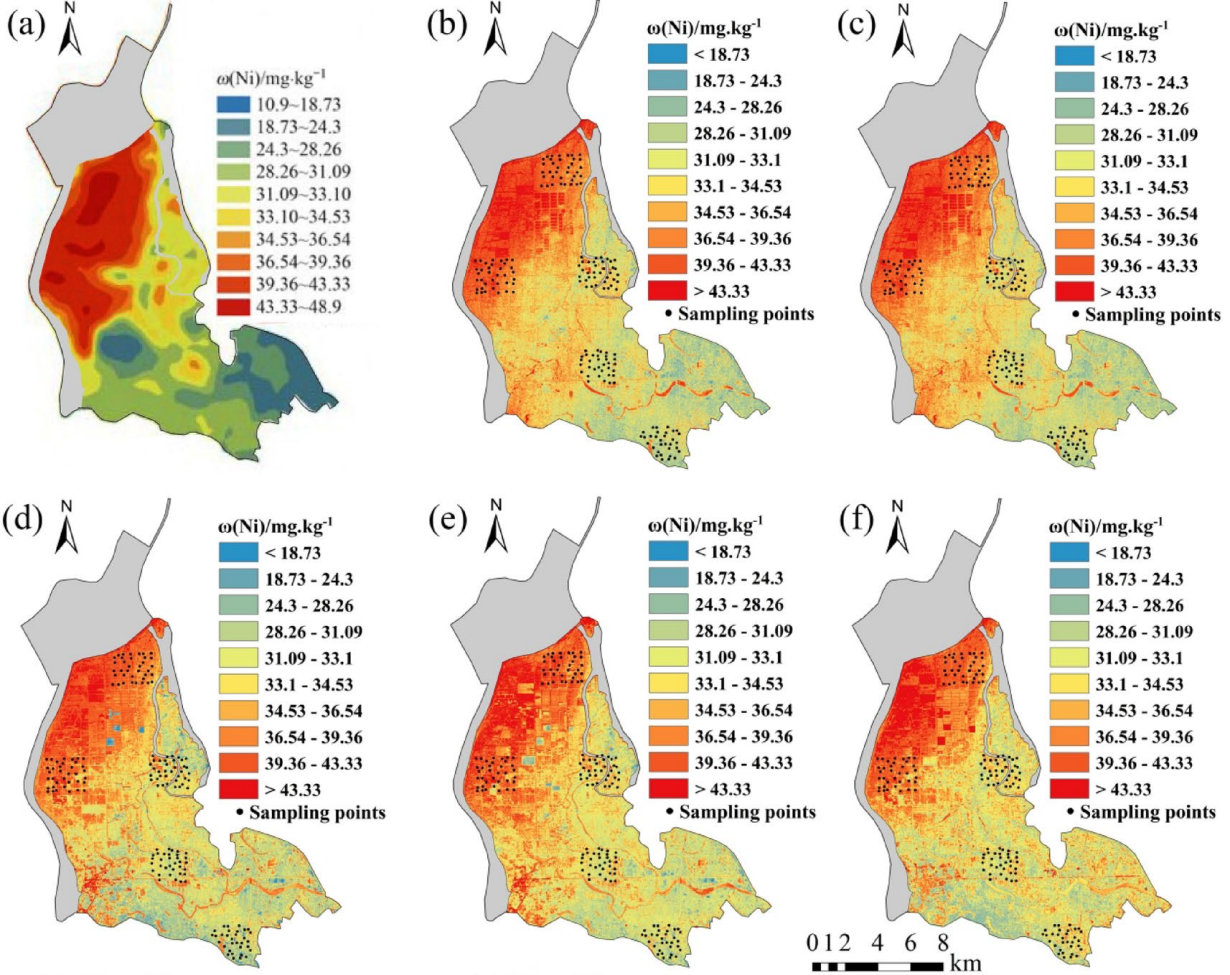
Soil Ni content distribution map: (**a**) Interpolation; (**b**) MLR; (**c**) PLSR; (**d**) SVM; (**e**) BP; (**f**) CNN.

**Fig. 12:**
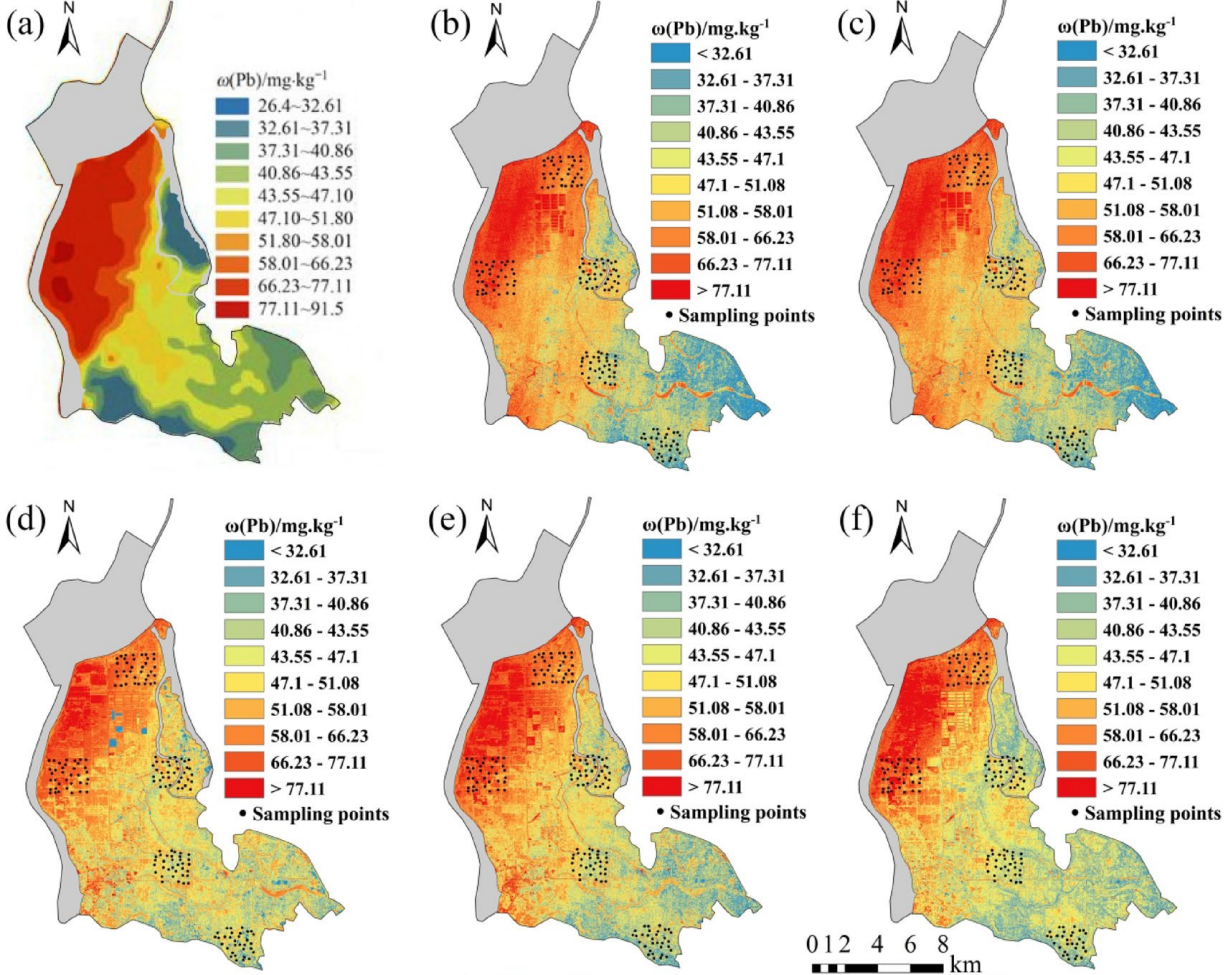
Soil Pb content distribution map: (**a**) Interpolation; (**b**) MLR; (**c**) PLSR; (**d**) SVM; (**e**) BP; (**f**) CNN.

**Fig. 13:**
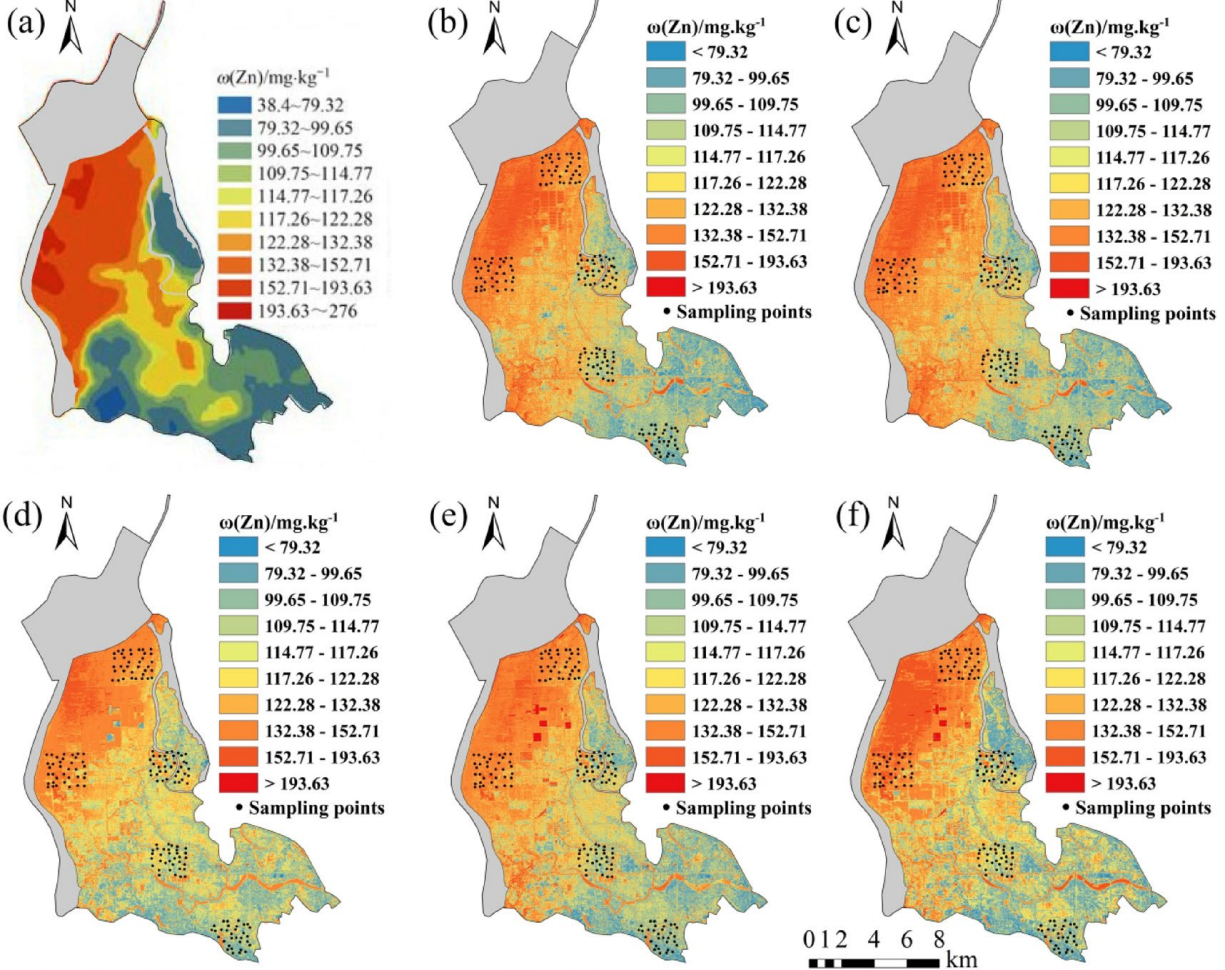
Soil Zn content distribution map: (**a**) Interpolation; (**b**) MLR; (**c**) PLSR; (**d**) SVM; (**e**) BP; (**f**) CNN.

Five regression models established for eight soil heavy metal elements were applied to the OHS image in the study area. The obtained spatial distribution map was compared with the spatial interpolation map of eight soil heavy metal contents obtained by Xiao et al.^32^ using the geochemical data of 1589 samples (Figs. 6, 7, 8, 9, 10, 11, 12 and 13). The 1589 sampling points were evenly distributed throughout the study area (Fig. 2). Since the sample points in this study are all located in the land area and do not include the sampling points in the water area, the water area is not considered when obtaining the spatial distribution map of soil heavy metal content.

The inversion results show that the soil heavy metal content in the Dongting Lake region is higher in the northwest and lower in the southeast, which is largely consistent with the results of the spatial interpolation map^32^ suggesting that the distribution of soil heavy metal content can be predicted by using OHS remote sensing image, and it is completely feasible to use a small number of samples to retrieve the distribution trends of heavy metals in large areas. Further analysis displays that the five models can basically predict the areas with high heavy metal content. However, the inversion accuracy of the other six soil heavy metal elements, except Hg and Zn, is poor in the areas with low content. Through the geographical location analysis of the sampling points, it is found that most of the six soil heavy metal elements in the sampling point areas belong to the areas with high heavy metal content. For example, the Cr content of the sampling point position is located in the high-value zone, where the Cr content of the inversion results is high, and the inversion accuracy is the worst in the low-value area. The content of As at the sampling point is mostly in the high-value areas. Although the test set inversion accuracy of As in the five inversion models is high, with an R^2^ value is larger than 0.7, the low-value zones of soil As content in the southeast corner of the study area still cannot be accurately predicted. The sampling points of Hg are evenly distributed in both the low-value and high-value areas with R^2^ of the test set of the five models being nearly 0.5–0.6. However, all five models showed low and high-value zones of soil Hg content.

## Discussion

Statistical analysis reveals that CNN and BP also perform poorly when the inversion accuracy of MLR and PLSR is low, such as in the retrieval of Cr and Cu. However, when the inversion accuracy of MLR and PLSR is high, CNN and BP usually perform better, the same as in the inversion of As, Cd, Ni, Pb, and other elements (Fig. 6). This may be due to the fact that when the spectral range of the image fails to cover the characteristic bands of the elements, even if more complex models such as CNN and BP are used, they cannot compensate for the missing information in the original data, and instead overfit the model to the training data, resulting in overfitting. When the inversion accuracy of MLR and PLSR is high, it indicates that there is a strong linear correlation between elements and bands. However, the relationship between soil elements and spectra is not simply linear, and there may be a complex nonlinear relationship. BP, CNN, etc. can learn both linear information and nonlinear information between elements and bands, which most likely improve the inversion accuracy of elements^29^.

Many previous studies have analyzed models that estimate soil heavy metal concentrations from OHS images (Table 6)^24,50–53^. In the retrieval of As, Cd, Ni, and Pb contents in soil, this study and the previous ones have achieved good retrieval accuracy, which further demonstrates that it is feasible to use OHS images to retrieve some heavy metal contents in soil. The results of Cd, Cr, Cu, and Pb in this paper are higher than the previous optimal results, which may be due to differences in soil and minerals in the study area. The mechanism of soil heavy metal enrichment is reflected in the adsorption of clay minerals, iron and manganese oxides, organic matter, and other substances^54–56^. Currently, heavy metal inversion studies use the spectral characteristics of heavy metal adsorbents to indirectly retrieve soil heavy metal content. The poor retrieval results of Cr and Cu in this paper are consistent with the results of previous studies, which may be due to the fact that the band range of the OHS image does not adequately cover the characteristic bands of Cr and Cu. Previous studies have shown that the characteristic bands of Cr are located near 1300 nm and 2200 nm, and the characteristic bands of Cu are located near 1800 nm and 2100nm^18,23,57,58^.

Healthy soils are essential for the successful implementation and achievement of the Sustainable Development Goals^58^. Using remote sensing data to quickly and accurately obtain the content of heavy metals in soil provides technical support for soil environmental protection and sustainable development. In this study, 175 samples from five sub-areas selected from 1589 samples distributed evenly in the study area were used to establish heavy metal inversion models, and compared with the heavy metal interpolation results using 1589 samples. The results show that it is feasible to use a small number of samples to invert the distribution trend of heavy metals in large areas. Although this study initially verified the feasibility of using a small number of samples to retrieve the distribution trend of heavy metals in a large area of soil, when the distribution of heavy metals in the sample was not uniform, the model results tended to the region where the heavy metal content in the sample was concentrated. How to solve this problem will also be the focus of our next research.

**Table 6:** R^2^ for soil heavy metal retrieval in the previous studies and this paper.

Element	*R*^2^ (This study)	Previous studies (OHS)		
		*R* ^2^	Model	Authors
As	0.686–0.740	0.780	PLSR	Xu et al. 2021^51^
Cd	0.544–0.651	0.136–0.650 and 0.80 (Adj R^2^)	MSR, PLSR	Liu 2021^50^; Xu et al. 2021^51^; Sun et al. 2024^53^
Cr	0.300–0.400	0.152–0.240 and 0.62 (Adj R^2^)	MSR, PLSR	Liu 2021^50^; Dai et al. 2022^52^; Sun et al. 2024^53^
Cu	0.212–0.381	0.124–0.324	MSR, PLSR	Liu 2021^50^; Dai et al. 2022^52^
Hg	0.463–0.528			
Ni	0.626–0.660	0.150–0.820	PLSR, RF, XGBoost, SVMR, GPR, BPNN	Xu et al. 2021^52^; Sun et al., 2023^24^
Pb	0.680–0.801	0.298–0.690	MSR, PLSR	Liu 2021^50^; Dai et al. 2022^52^
Zn	0.511–0.642			

## Conclusions

1. In this study, by integrating soil geochemical data and OHS remote sensing data, five regression models including MLR, PLSR, SVMR, BPNN and CNN were constructed, and high-precision prediction of heavy metal content such as As, Cd, Hg and Pb was successfully achieved. The results show that both linear and nonlinear models show good applicability in the inversion of specific elements, which confirms the technical feasibility of hyperspectral technology in large-scale soil heavy metal monitoring.
2. MLR and PLSR have the best inversion accuracy in Cr, Cu, Hg, Ni and Zn. The BPNN model shows the best stability and prediction accuracy in Pb element retrieval. The performance ability of the CNN model is particularly prominent for As and Cd elements. Therefore, there are significant differences in the sensitivity of different algorithms to heavy metal elements, and it is necessary to select an appropriate target model based on the characteristics of the target elements, which provides an algorithm optimization path for subsequent research.
3. This study has successfully reconstructed the large-scale distribution trends of heavy metals using a limited number of samples, which provides a method reference for soil monitoring under resource constraints.

## Data Availability

Due to the involvement of multiple interests in the data, it is not convenient to provide it directly. If you need to know more, please contact the corresponding author Dr. Gong Cheng directly.

## References

[1] Giller, K. E. & McGrath, S. P. Pollution by toxic metals on agricultural soils. *Nature* **335** (6192), 676. 10.1038/335676a0 (1988).

[2] Chen, T., Chang, Q., Clevers, J. G. P. W. & Kooistra, L. Rapid identification of soil cadmium pollution risk at regional scale based on visible and near-infrared spectroscopy. *Environ. Pollut.* **206**, 217–226. 10.1016/j.envpol.2015.07.009 (2015). 26188912 10.1016/j.envpol.2015.07.009

[3] Song, L. et al. Estimate of heavy metals in soil and streams using combined geochemistry and field spectroscopy in Wan-sheng mining area, chongqing, China. *Int. J. Appl. Earth Obs. Geoinf.* **34**, 1–9. 10.1016/j.jag.2014.06.013 (2015).

[4] Agomuo, E. N. & Amadi, P. U. Accumulation and toxicological risk assessments of heavy metals of top soils from markets in Owerri, Imo state, Nigeria. *Environmental nanotechnology, monitoring & management* **8**, 121–126. 10.1016/j.enmm.2017.07.001 (2017).

[5] Sun, H., Wan, S., Li, L. & Liu, D. Distribution of heavy metals in soil and plant of Reed wetland in the Dongting lake of China during different seasons. *J. Soil Water Conserv.* **29** (5), 289–293. 10.13870/j.cnki.stbcxb.2015.05.052 (2015).

[6] Fang, X. et al. Geochemistry of major and trace elements in sediments from inlets of the Xiangjiang and Yuanjiang river to Dongting lake, China. *Environ. Earth Sci.* **77**, 1–16. 10.1007/s12665-017-7193-5 (2018).

[7] Jiang, C. et al. Microplastics in Sediment and Surface Water of West Dongting Lake and South Dongting Lake: Abundance, Source and Composition. International Journal of Environmental Research and Public Health. **15 **(10), 2164 (2018). https://www.mdpi.com/1660-4601/15/10/2164 10.3390/ijerph15102164 PMC6210014 30275431

[8] Facchinelli, A., Sacchi, E. & Mallen, L. Multivariate statistical and GIS-based approach to identify heavy metal sources in soils. *Environ. Pollut*. **114** (3), 313–324. 10.1016/S0269-7491(00)00243-8 (2001). 11584630 10.1016/s0269-7491(00)00243-8

[9] Sun, G. X., Wang, X. J. & Hu, Q. H. Using stable lead isotopes to trace heavy metal contamination sources in sediments of Xiangjiang and Lishui rivers in China. *Environ. Pollut.* **159** (12), 3406–3410. 10.1016/j.envpol.2011.08.037 (2011). 21903315 10.1016/j.envpol.2011.08.037

[10] Chai, L. et al. Heavy metals and metalloids in the surface sediments of the Xiangjiang river, hunan, china: distribution, contamination, and ecological risk assessment. *Environ. Sci. Pollut. Res.* **24**, 874–885. 10.1007/s11356-016-7872-x (2017). 10.1007/s11356-016-7872-x 27761857

[11] Long, X. Estimation of Spatial distribution and health risk by arsenic and heavy metals in shallow groundwater around Dongting lake plain using GIS mapping. *Chemosphere* **269**, 128698. 10.1016/j.chemosphere.2020.128698 (2021). 33121802 10.1016/j.chemosphere.2020.128698

[12] Du Laing, G. Analysis and fractionation of trace elements in soils. *Trace Elem. soils*10.1002/9781444319477.ch4 (2010).

[13] D’Emilio, M., Macchiato, M., Ragosta, M. & Simoniello, T. A method for the integration of satellite vegetation activities observations and magnetic susceptibility measurements for monitoring heavy metals in soil. *J. Hazard. Mater.* **241**, 118–126. 10.1016/j.jhazmat.2012.09.021 (2012). 23044196 10.1016/j.jhazmat.2012.09.021

[14] Shi, T., Liu, H., Chen, Y., Wang, J. & Wu, G. Estimation of arsenic in agricultural soils using hyperspectral vegetation indices of rice. *J. Hazard. Mater.* **308**, 243–252. 10.1016/j.jhazmat.2016.01.022 (2016). 26844405 10.1016/j.jhazmat.2016.01.022

[15] Luce, M. S., Ziadi, N., Gagnon, B. & Karam, A. Visible near infrared reflectance spectroscopy prediction of soil heavy metal concentrations in paper mill biosolid-and liming by-product-amended agricultural soils. *Geoderma* **288**, 23–36. 10.1016/j.geoderma.2016.10.037 (2017).

[16] Dong, J., Dai, W., Xu, J. & Li, S. Spectral Estimation model construction of heavy metals in mining reclamation areas. *Int. J. Environ. Res. Public Health*. **13** (7), 640. 10.3390/ijerph13070640 (2016). 27367708 10.3390/ijerph13070640 PMC4962181

[17] Zhang, S. et al. Hyperspectral inversion of heavy metal content in reclaimed soil from a mining wasteland based on different spectral transformation and modeling methods. *Spectrochim. Acta Part A Mol. Biomol. Spectrosc.* **211**, 393–400. 10.1016/j.saa.2018.12.032 (2019). 10.1016/j.saa.2018.12.032 30594866

[18] Xue, Y., Zou, B., Wen, Y., Tu, Y. & Xiong, L. Hyperspectral inversion of chromium content in soil using support vector machine combined with lab and field spectra. *Sustainability* **12**, 4441. 10.3390/su12114441 (2020).

[19] Zhong, Q., Eziz, M., Ainiwaer, M. & Sawut, R. Hyperspectral inversion and analysis of zinc concentration in urban soil in the Urumqi City of China. *J. Environ. Earth Sci.* **5** (2), 76–87. 10.30564/jees.v5i2.5947 (2023).

[20] Zhou, Y. & Cheng, Y. Hyperspectral inversion of soil arsenic content in polymetallic mining areas based on optimized spectral index. *The Chinese Journal of Nonferrous Metals*10.11817/j.ysxb.1004.0609.2023-44331 (2024).

[21] Swain, R. & Sahoo, B. Mapping of heavy metal pollution in river water at daily time-scale using spatio-temporal fusion of MODIS-aqua and Landsat satellite imageries. *Hournal Environ. Manage.* **192**, 1–14. 10.1016/j.jenvman.2017.01.034 (2017). 10.1016/j.jenvman.2017.01.034 28130987

[22] Guan, Q. et al. Prediction of heavy metals in soils of an arid area based on multi-spectral data. *Joural Environ. Manage.* **243** (5), 137–143. 10.1016/j.jenvman.2019.04.109 (2019). 10.1016/j.jenvman.2019.04.109 31096168

[23] Lin, N. et al. Estimating the heavy metal contents in farmland soil from hyperspectral images based on stacked adaboost ensemble learning. *Ecol. Ind.* **143**, 109330. 10.1016/j.ecolind.2022.109330 (2022).

[24] Sun, Y., Chen, S., Dai, X., Jiang, H. & Jia, K. Coupled retrieval of heavy metal nickel concentration in agricultural soil from spaceborne hyperspectral imagery. *J. Hazard. Mater.* **446**, 130722. 10.1016/j.jhazmat.2023.130722 (2023). 36628862 10.1016/j.jhazmat.2023.130722

[25] Yang, L. et al. Estimating heavy metal concentrations in topsoil from vegetation reflectance spectra of Hyperion images: A case study of Yushu county, qinghai, China. *Chin. J. Appl. Ecol.* **27** (06), 1775–1784. 10.13287/j.1001-9332.201606.030 (2016). 10.13287/j.1001-9332.201606.030 29737683

[26] Yang, N., Han, L. & Liu, M. Inversion of soil heavy metals in metal tailings area based on different spectral transformation and modeling methods. *Heliyon* **9**(9), e19782. 10.1016/j.heliyon.2023.e19782 (2023). 37809479 10.1016/j.heliyon.2023.e19782 PMC10559111

[27] Wu, F. et al. Assessment of heavy metal pollution in agricultural soil around a gold mining area in Yitong county, china, based on satellite hyperspectral imagery. *J. Appl. Remote Sens.* **15** (4), 042613–042613. 10.1117/1.JRS.15.042613 (2021).

[28] Li, G. et al. Inversion of soil cd content using WorldView-3 multispectral and key environmental variables. *Trans. Chin. Soc. Agricultural Eng.* **38** (12), 224–232. 10.11975/j.issn.1002-6819.2022.12.026 (2022).

[29] Zhang, B. et al. Retrieving soil heavy metals concentrations based on GaoFen-5 hyperspectral satellite image at an opencast coal mine, inner mongolia, China. *Environ. Pollut.* **300**, 118981. 10.1016/j.envpol.2022.118981 (2022). 35150799 10.1016/j.envpol.2022.118981

[30] Zhao, H., Liu, P., Qiao, B. & Wu, K. The Spatial distribution and prediction of soil heavy metals based on measured samples and Multi-Spectral images in Tai lake of China. *Land* **10**, 1227. 10.3390/land10111227 (2021).

[31] Cheng, G. et al. Quantitative remote sensing of metallic elements for the Qishitan gold polymetallic mining area, NW China. *Remote Sens.* **13**, 2519. 10.3390/rs13132519 (2021).

[32] Xiao, K. Q. et al. Characteristics and source analysis of heavy metals in farmland soil on the South of Dongting lake. *Environ. Sci.* **44** (2), 932–943. 10.13227/j.hjkx.202203228 (2023). 10.13227/j.hjkx.202203228 36775616

[33] Zhang, R. et al. Simulation and assessment of the capabilities of Orbita hyperspectral (OHS) imagery for remotely monitoring Chlorophyll-a in eutrophic plateau lakes. *Remote Sens.* **13**, 2821. 10.3390/rs13142821 (2021).

[34] Jian, W., Yi, W., Wenlong, W., Lei, S. & Haiping, S. Transfer-learning-based cloud detection for Zhuhai-1 satellite hyperspectral imagery. *Front. Environ. Sci.* **10**, 1039249. 10.3389/fenvs.2022.1039249 (2022).

[35] Jiang, Y. et al. Geometric processing and accuracy verification of Zhuhai-1 hyperspectral satellites. *Remote Sens.* **11**, 996. 10.3390/rs11090996 (2019).

[36] Zhang, G. et al. Nickel grade inversion of lateritic nickel ore using WorldView-3 data incorporating Geospatial location information: A case study of North konawe, Indonesia. *Remote Sens.* **15**, 3660. 10.3390/rs15143660 (2023).

[37] Song, E. et al. Multi-Temporal remote sensing inversion of evapotranspiration in the lower Yangtze river based on Landsat 8 remote sensing data and analysis of driving factors. *Remote Sens.* **15**, 2887. 10.3390/rs15112887 (2023).

[38] Ma, F., Du, C., Zhou, J. & Shen, Y. Optimized self-adaptive model for assessment of soil organic matter using fourier transform mid-infrared photoacoustic spectroscopy. *Chemometr. Intell. Lab. Syst.* **171**, 9–15. 10.1016/j.chemolab.2017.09.017 (2017).

[39] Jia, P. et al. Inversion of different cultivated soil types’salinity using hyperspectral data and machine learning. *Remote Sens.* **14**, 5639. 10.3390/rs14225639 (2022).

[40] Jia, P., Shang, T., Zhang, J. & Sun, Y. Inversion of soil pH during the dry and wet seasons in the Yinbei region of ningxia, china, based on multi-source remote sensing data. *Geoderma Reg.* **25**, e00399. 10.1016/j.geodrs.2021.e00399 (2021).

[41] Tang, X. & Huang, M. Inversion of Chlorophyll-a concentration in Donghu lake based on machine learning algorithm. *Water* **13**, 1179. 10.3390/w13091179 (2021).

[42] Rumelhart, D. E., Hinton, G. E. & Williams, R. J. Learning representations by back-propagating errors. *Nature* **323** (6088), 533–536. 10.1038/323533a0 (1986).

[43] Liang, Y. J., Ren, C., Wang, H. Y., Huang, Y. B. & Zheng, Z. T. Research on soil moisture inversion method based on GA-BP neural network model. *Int. J. Remote Sens.* **40** (5–6), 2087–2103. 10.1080/01431161.2018.1484961 (2019).

[44] LeCun, Y., Bottou, L., Bengio, Y. & Haffner, P. Gradient-based learning applied to document recognition. *Proc. IEEE*. **86** (11), 2278–2324. 10.1109/5.726791 (1998).

[45] Wang, R., Zhao, J., Yang, H. & Li, N. Inversion of soil moisture on farmland areas based on SSA-CNN using Multi-Source remote sensing data. *Remote Sens.* **15** (10), 2515. 10.3390/rs15102515 (2023).

[46] Krizhevsky, A., Sutskever, I. & Hinton, G. E. ImageNet classification with deep convolutional neural networks. *Commun. ACM*. **60** (6), 84–90. 10.1145/3065386 (2017).

[47] Lu, C., Wang, Z., Wu, Z., Zheng, Y. & Liu, Y. Global ocean wind speed retrieval from GNSS reflectometry using CNN-LSTM network. *IEEE Trans. Geosci. Remote Sens.* **61**, 1–12. 10.1109/TGRS.2023.3276173 (2023).

[48] Sodango, T. H., Sha, J., Li, X. & Bao, Z. Assessment of machine-learning methods for the prediction of STN using multi-source data in Fuzhou city, *China Remote Sens. Applications: Soc. Environ.* **31**, 100995 10.1016/j.rsase.2023.100995. (2023).

[49] Zenhom, E. S., Nesma, A. A., Abdelaziz, L. A. & Youssef, M. Y. Machine learning-enhanced GALDIT modeling for the nile Delta aquifer vulnerability assessment in the mediterranean region. Groundwater for Sustainable Development, **28**, 101403 10.1016/j.gsd.2024.101403 (2025).

[50] Liu, S. Remote sensing retrieval of heavy metal content in farmland soil on Zhuhai-1 hyperspectral Data—Take an experimental field in Mianzhu City as an example. *Chengdu Univ. Technol.* (2021). https://link.oversea.cnki.net/doi/10.26986/d.cnki.gcdlc.2021.001190

[51] Xu, K., Wan, Y., Jiang, X. & Huang, B. Inversion Technology of Heavy Metal Pollution in Soil of Silong Town Based on OHS-D Data. *Environ Sci Technol* **44**(S1), 101–106. 10.19672/j.cnki.1003-6504.2021.S1.016 (2021).

[52] Dai, X. et al. Hyperspectral imagery reveals large Spatial variations of heavy metal content in agricultural soil-A case study of remote-sensing inversion based on Orbita hyperspectral satellites (OHS) imagery. *J. Clean. Prod.* **380**, 134878. 10.1016/j.jclepro.2022.134878 (2022).

[53] Sun, Y. et al. Towards interpretable machine learning for observational quantification of soil heavy metal concentrations under environmental constraints. *Sci. Total Environ.* **926**, 171931. 10.1016/j.scitotenv.2024.171931 (2024). 38531447 10.1016/j.scitotenv.2024.171931

[54] Garcıa-Sánchez, A., Alastuey, A. & Querol, X. Heavy metal adsorption by different minerals: application to the remediation of polluted soils. *Sci. Total Environ.* **242** (1–3), 179–188. 10.1016/S0048-9697(99)00383-6 (1999).

[55] Kemper, T. & Sommer, S. Estimate of heavy metal contamination in soils after a mining accident using reflectance spectroscopy. *Environ. Sci. Technol.* **36** (12), 2742–2747. 10.1021/es015747j (2002). 12099473 10.1021/es015747j

[56] Xiao, J. Y. et al. Review on methods of monitoring soil heavy metal based on hyperspectral remote sensing data. *Hubei Agricultural Sci.* **52** (6), 1248–1253. 10.14088/j.cnki.issn0439-8114.2013.06.019 (2013).

[57] Liu, J., Zhang, Y., Wang, H. & Du, Y. Study on the prediction of soil heavy metal elements content based on visible near-infrared spectroscopy. *Spectrochim. Acta Part A Mol. Biomol. Spectrosc.* **199**, 43–49. 10.1016/j.saa.2018.03.040 (2018). 10.1016/j.saa.2018.03.040 29562213

[58] Mamat, S., Abudugheni, A. & Hu, X. Hyperspectral inversion study of heavy metals content in soils of oasis farmland in arid region. *China Environ. Sci.*10.19674/j.cnki.issn1000-6923.20231128.008 (2023).

